# Statistical sensitivity for detection of spatial and temporal patterns in rodent population densities.

**DOI:** 10.3201/eid0501.990114

**Published:** 1999

**Authors:** C A Parmenter, T L Yates, R R Parmenter, J L Dunnum

**Affiliations:** Department of Biology, University of New Mexico, Albuquerque 87131-1091, USA. cparment@unm.edu

## Abstract

A long-term monitoring program begun 1 year after the epidemic of hantavirus pulmonary syndrome in the U.S. Southwest tracked rodent density changes through time and among sites and related these changes to hantavirus infection rates in various small-mammal reservoir species and human disease outbreaks. We assessed the statistical sensitivity of the program's field design and tested for potential biases in population estimates due to unintended deaths of rodents. Analyzing data from two sites in New Mexico from 1994 to 1998, we found that for many species of Peromyscus, Reithrodontomys, Neotoma, Dipodomys, and Perognathus, the monitoring program detected species-specific spatial and temporal differences in rodent densities; trap-related deaths did not significantly affect long-term population estimates. The program also detected a short-term increase in rodent densities in the winter of 1997-98, demonstrating its usefulness in identifying conditions conducive to increased risk for human disease.


### Statistical Sensitivity for Detection of Spatial and Temporal Patterns in Rodent Population Densities

**Cheryl A. Parmenter, Terry L. Yates, Robert R. Parmenter, and Jonathan L. Dunnum**
University of New Mexico, Albuquerque, New Mexico, USA

A long-term monitoring program begun 1 year after the epidemic of hantavirus pulmonary syndrome in the U.S. Southwest tracked rodent density changes through time and among sites and related these changes to hantavirus infection rates in various small-mammal reservoir species and human disease outbreaks. We assessed the statistical sensitivity of the program's field design and tested for potential biases in population estimates due to unintended deaths of rodents. Analyzing data from two sites in New Mexico from 1994 to 1998, we found that for many species of *Peromyscus, Reithrodontomys, Neotoma, Dipodomys*, and *Perognathus*, the monitoring program detected species-specific spatial and temporal differences in rodent densities; trap-related deaths did not significantly affect long-term population estimates. The program also detected a short-term increase in rodent densities in the winter of 1997-98, demonstrating its usefulness in identifying conditions conducive to increased risk for human disease.

We analyzed the statistical capabilities of the long-term rodent monitoring program begun in 1994 to detect spatial and temporal changes in rodent densities and determine if the low death rates at all study sites resulted in biased (underestimated) rodent population density estimates. We also examined a short-term subset of the data (mid-1997 to early 1998) to test whether the program design could statistically detect a sudden rodent increase in density that may precede a hantavirus pulmonary syndrome outbreak.

### Study Sites

We selected two monitoring sites in New Mexico for analysis because they provided the longest period of field sampling, the greatest range in rodent species richness, and the largest difference in habitat types. The first, a desert grassland site 90 km south of Albuquerque, on the Sevilleta National Wildlife Refuge, Socorro County, New Mexico, USA (34º 21.3'N, 106º 53.1'W, elevation 1,465 m), was dominated by one-seed juniper (*Juniperus monosperma*), honey mesquite (*Prosopis glandulosa*), and various desert grasses (*Sporobolus* spp. and *Bouteloua eriopoda*). The second site (Placitas), a pinyon-juniper woodland site located in Sandoval County in the foothills of the Sandia Mountains, Cibola National Forest, approximately 30 km north of Albuquerque, New Mexico, USA (35º 16.7'N, 106º 18.6'W, elevation 1,830 m), was dominated by pinyon pine (*Pinus edulis*), one-seed juniper, and blue grama grass (*Bouteloua gracilis*).

### Statistical Methods

The overall experimental design and field sampling procedures are described by Mills et al. (this issue, pp. 95-101). The rodent density estimates we analyzed were based on mark-recapture trapping data from three permanently marked trapping webs at each site (1,2). We used monthly trapping data collected from August 1994 to February 1998. Trapping and rodent handling methods were described by Parmenter et al. (3), and safety procedures during animal processing followed Mills et al. (4). Blood samples collected from *Peromyscus maniculatus* were analyzed by the Centers for Disease Control and Prevention for Sin Nombre virus (SNV). Rodent densities were calculated by the program DISTANCE (2). Each dataset was analyzed by three models (uniform, half-normal, and hazard)

Address for correspondence: Cheryl A. Parmenter, Department of Biology, The University of New Mexico, 167 Castetter Hall, Albuquerque, NM 87131-1091, USA; fax: 505-277-0304; e-mail: cparment@unm.edu.

with three possible model adjustments (cosine, polynomial, and hermite). Akaike's information criterion was then used to select the model that best fit the particular dataset (2). The three web density estimates for each trapping period were then partitioned into "proportional" densities representing each species, on the basis of the relative proportion of each in the total web sample. These species-specific densities (in numbers of mice per hectare) were analyzed by a repeated measures analysis of variance (RMANOVA) to test for differences between sites and through time and for site and time interactions. If significant F values were observed, we conducted within-trapping-period tests to detect differences among site means; we used Fisher's least-significant-difference method.

The effect of low death rates among rodents on estimates of population size was analyzed by minimum number alive (MNA) methods (5). For this analysis, we calculated the "observed" MNA values for each species during each sampling period on the basis of actual field data, which included occasional trap deaths of rodents. We then constructed a hypothetical MNA, assuming that no trap deaths occurred in the sampled populations. The hypothetical death-free MNAs were computed by extending the projected life span of each animal that died in the trap. The length of the extensions differed by species and site and was determined by the mean number of trapping periods during which each species would normally have been present on each site (this figure was based on the lifespans of all other mice of that species that did not die in the traps or during handling). For example, if *Neotoma albigula* at the Sevilleta National Wildlife Refuge site, Web 1, had a mean lifespan of three trapping periods (i.e., its initial capture period [time zero] plus three additional sampling periods), and a mouse died in a trap on its first capture, we would add one mouse to the observed MNA values for three additional trapping periods to arrive at the hypothetical MNA values. If the mouse died during its second trapping period, we would add two trapping periods to the MNA estimates, and so on. In addition, if the dead mouse was pregnant or lactating, we increased the hypothetical MNA by the average number of offspring that would have been produced; mean numbers of offspring for each species were determined from specimen databases at the University of New Mexico's Museum of Southwestern Biology. These offspring were included for the duration of the expected lifespan on each study site. Thus, if a pregnant female *N. albigula* died during the study, we would add two offspring (the mean litter size for this species in New Mexico) to the MNA estimates for the full three trapping periods of their life expectancy. This process created species-specific hypothetical MNA values that were either equal to or greater than the observed MNA values. The two MNA datasets were then compared by RMANOVA.

### Spatial Differences in Rodent Densities

RMANOVA successfully distinguished rodent densities between sites for a number of species (Table 1). Ord's kangaroo rat and the

Table 1. Repeated-measures analysis of variance testing for differences among representative rodent population densities[a], Sevilleta National Wildlife Refuge and Placitas, 1994-1998

| Species | Source | DF | F value | p |
|---|---|---|---|---|
| *Dipodomys ordii* | Site | 1 | 7.52 | 0.0517 |
| | Error (Site) | 4 | | |
| (Ord's kangaroo rat) | Time | 35 | 2.19 | 0.0007 |
| | Time x Site | 35 | 2.21 | 0.0006 |
| | Error (Time) | 140 | | |
| *Perognathus flavescens* | Site | 1 | 25.69 | 0.0071 |
| | Error (Site) | 4 | | |
| (Plains pocket mouse) | Time | 35 | 2.91 | 0.0001 |
| | Time x Site | 35 | 2.89 | 0.0001 |
| | Error (Time) | 140 | | |
| *Peromyscus maniculatus* | Site | 1 | 0.28 | 0.6233 |
| | Error (Site) | 4 | | |
| (Deer mouse) | Time | 35 | 2.80 | 0.0001 |
| | Time x Site | 35 | 1.18 | 0.2513 |
| | Error (Time) | 140 | | |
| *Peromyscus leucopus* | Site | 1 | 0.11 | 0.7609 |
| | Error (Site) | 4 | | |
| (White-footed mouse) | Time | 35 | 7.25 | 0.0001 |
| | Time x Site | 35 | 4.56 | 0.0001 |
| | Error (Time) | 140 | | |
| *Peromyscus truei* (Pinyon mouse) | Site | 1 | 1.98 | 0.2328 |
| | Error (Site) | 4 | | |
| | Time | 35 | 3.77 | 0.0001 |
| | Time x Site | 35 | 3.29 | 0.0001 |
| | Error (Time) | 140 | | |
| *Neotoma albigula* | Site | 1 | 6.65 | 0.0614 |
| | Error (Site) | 4 | | |
| (White-throated wood rat) | Time | 35 | 1.53 | 0.0431 |
| | Time x Site | 35 | 1.65 | 0.0221 |
| | Error (Time) | 140 | | |
| *Reithrodontomys megalotis* | Site | 1 | 0.34 | 0.5906 |
| | Error (Site) | 4 | | |
| (Western harvest mouse) | Time | 35 | 4.04 | 0.0001 |
| | Time x Site | 35 | 3.57 | 0.0001 |
| | Error (Time) | 140 | | |

[a]Density=number of mice per hectare.

Plains pocket mouse (Heteromyidae) were much more abundant at the Sevilleta National Wildlife Refuge site than at Placitas (Figure 1A, B) and had greater statistical differences in the RMANOVA results (Table 1). In contrast, other rodent species (Muridae) had no overall differences by site (Table 1) and usually had similar densities, except for occasional episodes (Figures 1C-G). Although we observed no overall effect of site on density for these species, Fisher's least-significant-difference methods showed significant differences between sites during certain periods (Figures 1C,D,F,G), demonstrating that within a particular species, intersite differences could be discerned in both long-term sequences and during episodic, site-specific population irruptions.

### Temporal Changes in Rodent Densities

The analyses also detected changes in rodent densities through time in all species examined (Table 1). Several species with generally low densities (e.g., the harvest mouse [Figure 1D], the white-footed mouse [Figure 1E], and the deer mouse [Figure 1G]) occasionally became locally extinct but periodically recolonized the sites. Other species (e.g., the pinyon mouse [Figure 1F] and the white-throated wood rat [Figure 1C]) were found consistently on both sites, although their densities fluctuated considerably. In all cases, RMANOVA found significant differences in these temporal patterns.

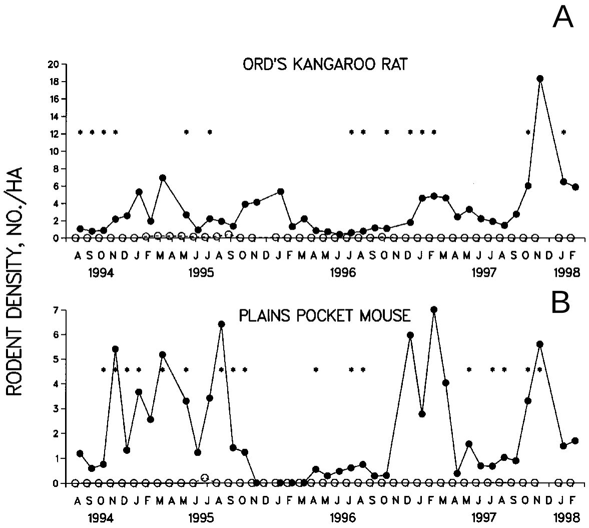

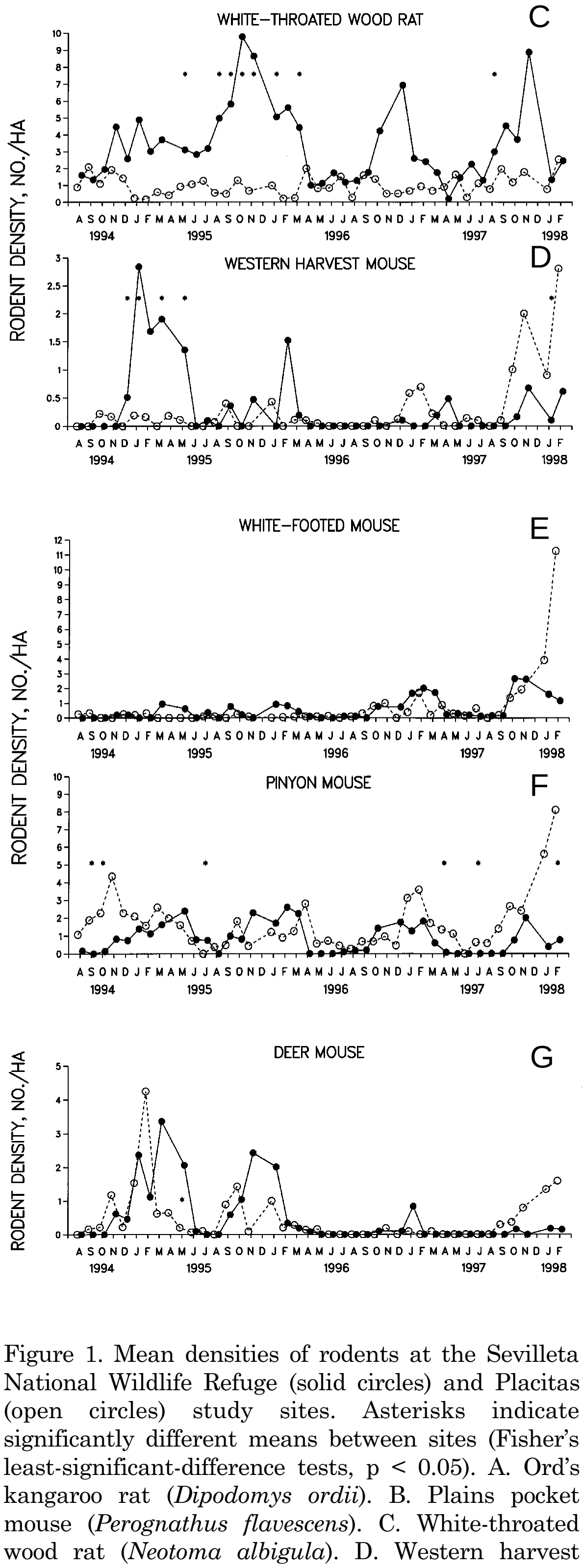

Figure 1. Mean densities of rodents at the Sevilleta National Wildlife Refuge (solid circles) and Placitas (open circles) study sites. Asterisks indicate significantly different means between sites (Fisher's least-significant-difference tests, $p < 0.05$). A. Ord's kangaroo rat (*Dipodomys ordii*). B. Plains pocket mouse (*Perognathus flavescens*). C. White-throated wood rat (*Neotoma albigula*). D. Western harvest mouse (*Reithrodontomys megalotis*). E. White-footed mouse (*Peromyscus leucopus*). F. Pinyon mouse (*P. truei*). G. Deer mouse (*Peromyscus maniculatus*).

### Short-Term Rodent Population Increases

To determine the capability of the analyses to show statistically significant short-term increases in rodent densities (e.g., a rodent population "explosion"), we selected May 1997 to February 1998, a period characterized by a rodent density increase in some species (Figures 1A-G). We then tested the datasets for this period alone; RMANOVA results indicated significant increases in densities for the Plains pocket mouse, deer mouse, white-footed mouse, pinyon mouse, and Western harvest mouse (Table 2; Figure 1 B,D-G) during the winter of 1997 to 1998. Therefore, the monitoring program was capable of showing sudden, short-term increases in rodent densities that may precede a disease outbreak in humans.

Blood tests to determine the presence of SNV in deer mice showed generally low infection rates (Figure 2), with a maximum of only one mouse testing positive per trapping period at Placitas, and none at Sevilleta. The SNV-positive rodents were detected during periods of moderate abundance in 1994 and 1995 but not in the early stage of the population increase during the winter of 1997-98.

Table 2. Short-term repeated-measures analysis of variance for differences among representative rodent population densities, Sevilleta National Wildlife Refuge and Placitas , May 1997 to February 1998

| Species | Source | DF | F value | p |
|---|---|---|---|---|
| *Dipodomys ordii* | Site | 1 | 5.75 | 0.0745 |
| | Error (Site) | 4 | | |
| (Ord's kangaroo rat) | Time | 8 | 2.13 | 0.0616 |
| | Time x Site | 8 | 2.13 | 0.0616 |
| | Error (Time) | 32 | | |
| *Perognathus flavescens* | Site | 1 | 27.09 | 0.0065 |
| | Error (Site) | 4 | | |
| (Plains pocket mouse) | Time | 8 | 4.61 | 0.0008 |
| | Time x Site | 8 | 4.61 | 0.0008 |
| | Error (Time) | 32 | | |
| *Peromyscus maniculatus* | Site | 1 | 2.70 | 0.1759 |
| | Error (Site) | 4 | | |
| (Deer mouse) | Time | 35 | 2.45 | 0.0432 |
| | Time x Site | 35 | 1.57 | 0.1868 |
| | Error (Time) | 140 | | |
| *Peromyscus leucopus* | Site | 1 | 2.25 | 0.2084 |
| | Error (Site) | 4 | | |
| (White-footed mouse) | Time | 8 | 7.83 | 0.0001 |
| | Time x Site | 8 | 5.79 | 0.0001 |
| | Error (Time) | 32 | | |
| *Peromyscus truei* | Site | 1 | 5.09 | 0.0870 |
| | Error (Site) | 4 | | |
| (Pinyon mouse) | Time | 8 | 7.09 | 0.0001 |
| | Time x Site | 8 | 4.94 | 0.0005 |
| | Error (Time) | 32 | | |
| *Neotoma albigula* | Site | 1 | 1.06 | 0.3618 |
| | Error (Site) | 4 | | |
| (White-throated wood rat) | Time | 8 | 1.17 | 0.3443 |
| | Time x Site | 8 | 0.82 | 0.5944 |
| | Error (Time) | 32 | | |
| *Reithrodontomys megalotis* | Site | 1 | 4.58 | 0.0990 |
| | Error (Site) | 4 | | |
| (Western harvest mouse) | Time | 8 | 4.67 | 0.0007 |
| | Time x Site | 8 | 1.64 | 0.1530 |
| | Error (Time) | 32 | | |

Density = numbers of mice per hectare.

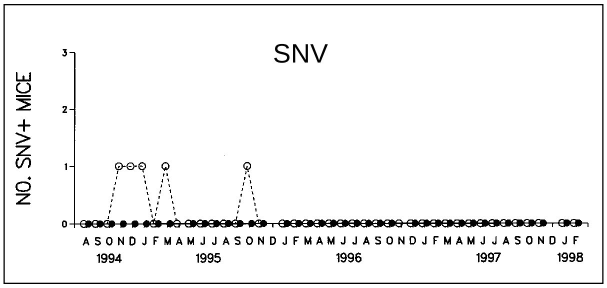

Figure 2. Seroprevalence of Sin Nombre virus (SNV) in the deer mouse populations at Sevilleta National Wildlife Refuge and Placitas study sites.

### Low Death Rates among Rodents and Population Estimates

Four species had sufficient sample sizes for the MNA analyses, which were based on 28 trapping periods between August 1994 and January 1997 and included 7,024 rodent captures (Table 3). During field sampling, trap death rates were generally lower than 10% (3). A breakdown of the number of new animals, recaptured resident animals, and trap deaths indicated that immigration and reproduction rates were consistently higher than trap death rates (Figure 3A-D). Species showing territorial behavior (e.g., Merriam's kangaroo rat [Figure 3A] and the white-throated wood rat [Figure 3C]) had higher ratios of residents to immigrants than species without strongly defended territories.

In constructing the hypothetical MNA values, we used the following mean life expectancy values (number of trapping periods after initial capture): *D. merriami* = 2.16; *P. flavescens* = 1.11; *P. truei* = 0.68; *N. albigula* = 2.75. For female rodents of reproductive age, the following mean numbers of offspring were used (on the basis of University of New Mexico's

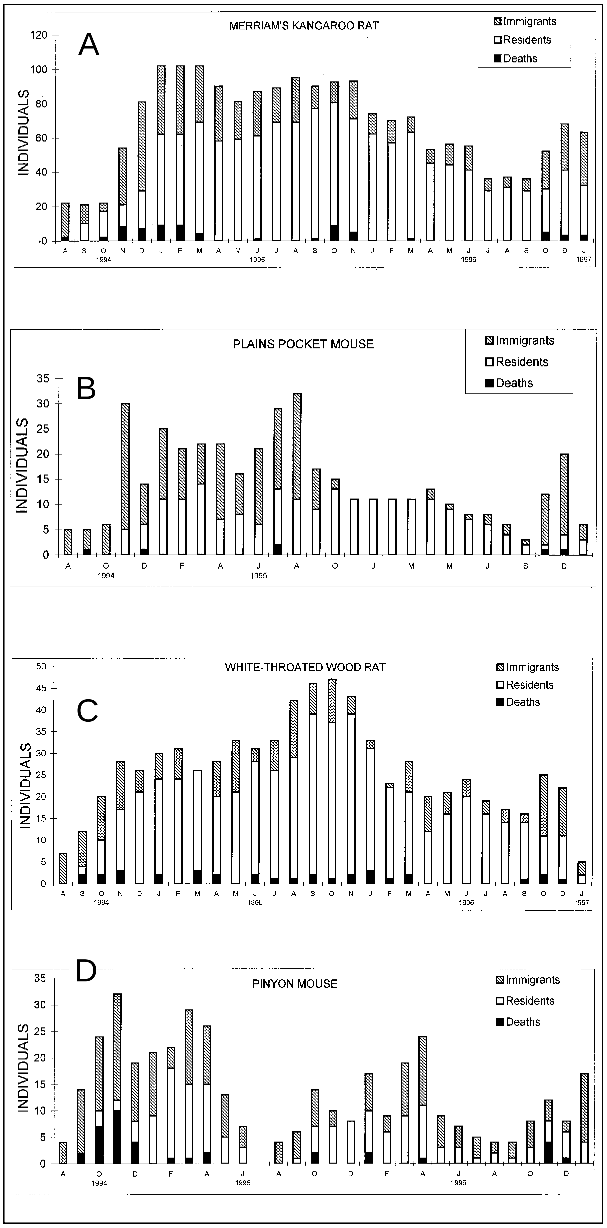

Figure 3. Composition of sampled populations at the Sevilleta National Wildlife Refuge study site. The number of immigrants greatly exceeds incidental trap deaths. A. Merriam's kangaroo rat (*Dipodomys merriami*). B. Plains pocket mouse (*Perognathus flavescens*). C. White-throated wood rat (*Neotoma albigula*). D. Pinyon mouse (*Peromyscus truei*).

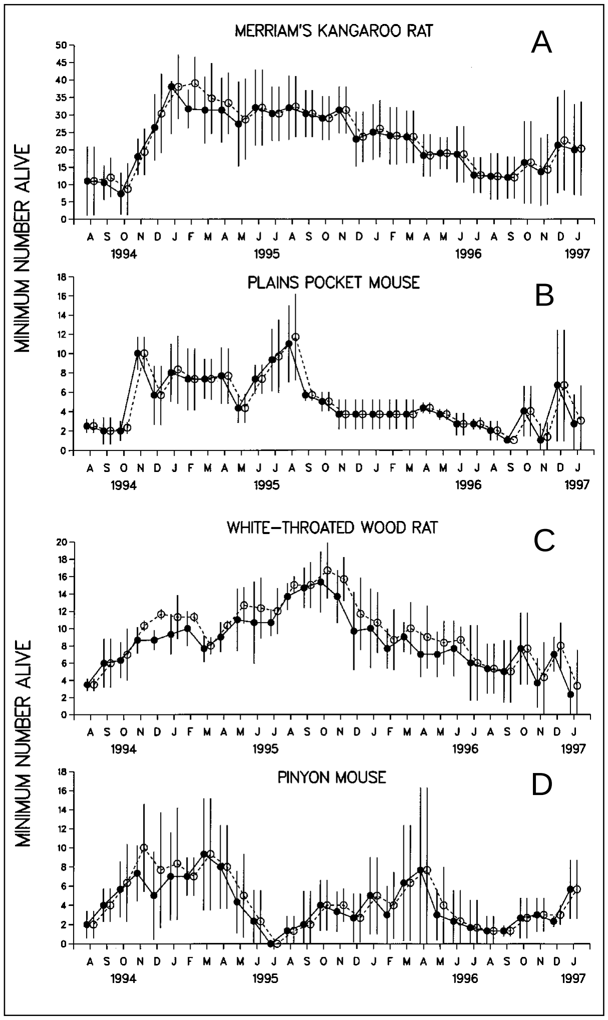

Figure 4. Mean minimum number alive (MNA) values of "observed" (solid circles) and "hypothetical" (open circles) for the following:

A. Merriam's kangaroo rat *(Dipodomys merriami)*

B. Plains pocket mouse (*Perognathus flavescens*)

C. White-throated wood rat (*Neotoma albigula*) at the Sevilleta National Wildlife Refuge study site

D. Pinyon mouse (*Peromyscus truei*) at the Placitas study site.

Values are means of three replicate trapping sites; error bars represent one standard deviation.

Table 3. Repeated-measures analysis of variance for differences between actual minimum number alive (MNA) estimates and hypothetical MNAs (zero deaths from trapping), New Mexico, 1994 to 1997

| Species | Source | DF | F value | p |
|---|---|---|---|---|
| *Dipodomys merriami* | MNA Treatment | 1 | 0.03 | 0.8672 |
| (Merriam's | Time | 27 | 21.19 | 0.0001 |
| kangaroo | Time x MNA | 27 | 0.30 | 0.9997 |
| rat) | Error (Time) | 108 | | |
| *Perognathus* | MNA treatment | 1 | 0.08 | 0.7858 |
| *flavescens* | Error (MNA) | 4 | | |
| (Plains | Time | 27 | 7.99 | 0.0001 |
| pocket | Time x MNA | 27 | 0.01 | 1.0000 |
| mouse) | Error (Time) | 108 | | |
| *Peromyscus* | MNA treatment | 1 | 0.03 | 0.8661 |
| *truei* | Error (MNA) | 4 | | |
| (Pinyon | Time | 27 | 6.24 | 0.0001 |
| mouse) | Time x MNA | 27 | 0.13 | 1.0000 |
| | Error (Time) | 108 | | |
| *Neotoma* | MNA treatment | 1 | 0.57 | 0.4914 |
| *albigula* | Error (MNA) | 4 | | |
| (White- | Time | 27 | 11.82 | 0.0001 |
| throated | Time x MNA | 27 | 0.15 | 1.0000 |
| wood rat) | Error (Time) | 108 | | |

Museum of Southwestern Biology data): *D. merriami* = 2.03; *P. flavescens* = 2.75; *P. truei* = 3.46; *N. albigula* = 1.83. When analyzed with RMANOVA, observed MNA values were not different from hypothetical (Table 3). While highly significant temporal differences in MNA values were observed (Figures 4A-D), no treatment-by-time interactions were produced, which demonstrated that the low death rates during the monitoring program did not affect rodent population estimates.

### Conclusions

Our analyses indicated that the field experimental statistical design was sufficiently sensitive to detect a range of differences in densities of rodent species across study sites and through time. Even relatively moderate levels of increases were detectable by our methods, although they were far less dramatic than those observed during the 1993 SNV outbreak (6). While rodent densities of certain species significantly increased during this study (1994 to 1998), the maximum densities were considerably lower than those observed at the Sevilleta National Wildlife Refuge site in 1993 during the SNV outbreak (6). In addition, seroprevalence in deer mice dropped to zero in 1996 (Figure 1G) and did not return, despite the higher densities observed in this species at these sites. Clearly, the population dynamics observed at this time were not equivalent to the rodent "outbreak" of 1993. Continued monitoring of these populations will be needed to determine the extent and importance of this apparent trend in rodent population ecology.

The statistical sensitivity demonstrated in this study is critical to the success of field monitoring programs, particularly those that function as early warning systems to alert health-care workers and researchers of impending outbreaks of disease (7,8). In the case of SNV, the monitoring program serves as both an early warning system and as a research database to which numerous environmental variables and the prevalence of SNV infections in rodents can be correlated.

In addition to the need for statistical power in distinguishing spatial and temporal patterns, the monitoring program must provide study site population estimates that can be directly compared. Standardization of techniques used by collaborating research groups ensures such comparability. In these hantavirus studies, the use of trapping webs and distance sampling theory (2) also allows for direct comparisons of rodent densities among species and across widely varying ecosystems. Trapping grids, and their associated population estimators, often produce results that may be suitable for local studies (internally consistent within a single experiment), but cannot be compared across ecosystem types and taxa because of site-specific characteristics or species-specific assumptions of capture probabilities. Trapping webs and distance sampling density estimators, however, have produced reasonably accurate density estimates in both a computer simulation study (9) and a field study (10). The accuracy of trapping webs and grids in estimating rodent densities is being evaluated more fully at the Sevilleta National Wildlife Refuge site.

All these methods require an appropriate sampling design and an understanding of the basic biology of the rodents. For example, the Plains pocket mouse has MNA values of four animals per month for the months of November, 1994 to March 1995 (Figure 4B). In contrast, its density for the same period is zero (Figure 1B), which indicates that no animals were captured during field sampling. The species' habit of becoming inactive and remaining in underground burrows during cold winter weather accounts for this discrepancy. Thus, in ecologic and perhaps disease transmission terms, density data accurately reflect which species of rodents are active on a site, whereas MNA data show the combined survivorship of active and inactive species.

A potential source of estimation bias in rodent monitoring programs is the inadvertent influence of trapping and handling of rodents during field sampling. Capturing, anesthetizing, measuring, and collecting blood and saliva samples traumatizes small animals and may affect future trapping success, which, in turn, could bias the accuracy of the density or population estimators. Previous studies have shown various effects of trapping and handling on rodent body mass, ability to trap rodents in the future, and survival (3,11,12-17), but none have addressed the effect of low death rates on long-term population trends. While precautions are taken to ensure survival of sampled animals, occasionally a few die during sampling, especially those with a lower tolerance to the physical, physiologic, and psychologic stress of being captured and handled. Chronic loss of study animals from trapping or handling could underestimate their densities when compared to those of "natural" or undisturbed populations nearby. Results of our study indicate that death rates from trapping at these sites had no significant effect on long-term rodent population estimates.

The existing network of rodent population study sites seems successful in identifying local species-specific fluctuations in densities. Data from these sites can be used in addressing hypotheses on the relationships among environmental factors, rodent abundance, and SNV infections, as well as in providing an early warning for potential rodent population explosions that may increase the risk for other hantavirus pulmonary syndrome outbreaks in the southwestern United States.

### Acknowledgments

We thank the U.S. Fish and Wildlife Service (Sevilleta National Wildlife Refuge) and the U.S. Forest Service (Cibola National Forest) for their cooperation in the study.

This research was supported by a collaborative agreement between the Department of Biology, University of New Mexico, the U.S. Centers for Disease Control and Prevention, the Indian Health Service, and the State of New Mexico Health Department. Additional support was provided by the Sevilleta Long-Term Ecological Research Program (NSF Grant DEB-9411976), the Museum of Southwestern Biology, and the National Institutes of Health (Grant 1 PO1 AI39780).

Cheryl A. Parmenter is a research associate in the University of New Mexico's Museum of Southwestern Biology and data manager for the New Mexico Hantavirus Monitoring Team.

### References

1. Anderson DR, Burnham KP, White GC, Otis DL. Density estimation of small-mammal populations using a trapping web and distance sampling methods. Ecology 1983;64:674-80.
2. Buckland ST, Anderson DR, Burnham KP, Laake JL. Distance sampling. Estimating abundance of biological populations. New York: Chapman and Hall, 1993.
3. Parmenter CA, Yates TL, Parmenter RR, Mills JN, Childs JE, Campbell ML, et al. Small mammal survival and trapability in mark-recapture monitoring programs for hantavirus. J Wildl Dis 1998;34:1-12.
4. Mills JN, Yates TL, Childs JE, Parmenter RR, Ksiazek TG, Rollin PE, et al. Guidelines for working with rodents potentially infected with hantavirus. Journal of Mammalogy 1995;76:716-22.
5. Krebbs CJ. Demographic changes in fluctuating populations of *Microtus californicus*. Ecological Monographs 1966;36:239-73.
6. Parmenter RR, Brunt JA, Moore DL, Ernest SM. The hantavirus epidemic in the Southwest: rodent population dynamics and the implications for transmission of Hantavirus-associated adult respiratory distress syndrome (HARDS) in the Four Corners region. Report to the Federal Centers for Disease Control and Prevention, July 1993. Publication No. 41. Sevilleta Long-Term Ecological Research Program (LTER) 1993:1-45.
7. Mills JN, Ellis BA, McKee KT Jr, Calderon GE, Maiztegui JI, Nelson GO, et al. A longitudinal study of Junin virus activity in the rodent reservoir of Argentine hemorrhagic fever. Am J Trop Med Hyg 1992;47:749-63.
8. Mills JN, Ellis BA, Childs JE, McKee KT Jr, Maiztegui JI, Peters CJ, et al. Prevalence of infection with the Junin virus in rodent populations in the epidemic area of Argentine hemorrhagic fever. Am J Trop Med Hyg 1994;51:554-62.
9. Wilson KR, Anderson DR. Evaluation of a density estimator on a trapping web and distance sampling theory. Ecology 1985;66:1185-94.
10. Parmenter RR, MacMahon JA, Anderson DR. Animal density estimation using a trapping web design: field validation experiments. Ecology 1989;70:169-79.
11. Swann DE, Kuenzi AJ, Morrison ML, DeStefano S. Effects of sampling blood on survival of small mammals. Journal of Mammalogy 1997;78:908-13.

12. Slade NA, Iskjaer C. Daily variation in body mass of free-living rodents and its significance for mass-based population dynamics. Journal of Mammalogy 1990;71:357-63.
13. Wood MD, Slade NA. Comparison of ear-tagging and toe-clipping in prairie voles, *Microtus ochrogaster*. Journal of Mammalogy 1990;71:252-5.
14. Slade NA. Loss of body mass associated with capture of *Sigmodon* and *Microtus* from northeastern Kansas. Journal of Mammalogy 1991;72:171-6.
15. Kaufman GA, Kaufman DW. Changes in body mass related to capture in the prairie deer mouse (*Peromyscus maniculatus*). Journal of Mammalogy 1994;75:681-91.
16. Douglass RJ, Van Horn R, Coffin KW, Zanto SN. Hantavirus in Montana deer mouse populations: Preliminary results. J Wildl Dis 1996;23:527-30.
17. Vanblankenstein T, Botzler RG. Effect of ectoparasite removal procedures on recapture of *Microtus californicus*. J Wildl Dis 1996;32:714-5.
